# Supplementary material for: Structural insights into two distinct binding modules for Lys63-linked polyubiquitin chains in RNF168
Source: Nat Commun. 2018 Jan 12;9:170. doi: 10.1038/s41467-017-02345-y (PMC5766498; doi:10.1038/s41467-017-02345-y)
Supplement: Supplementary file 1 — Supplementary Information [file 41467_2017_2345_MOESM1_ESM.pdf]

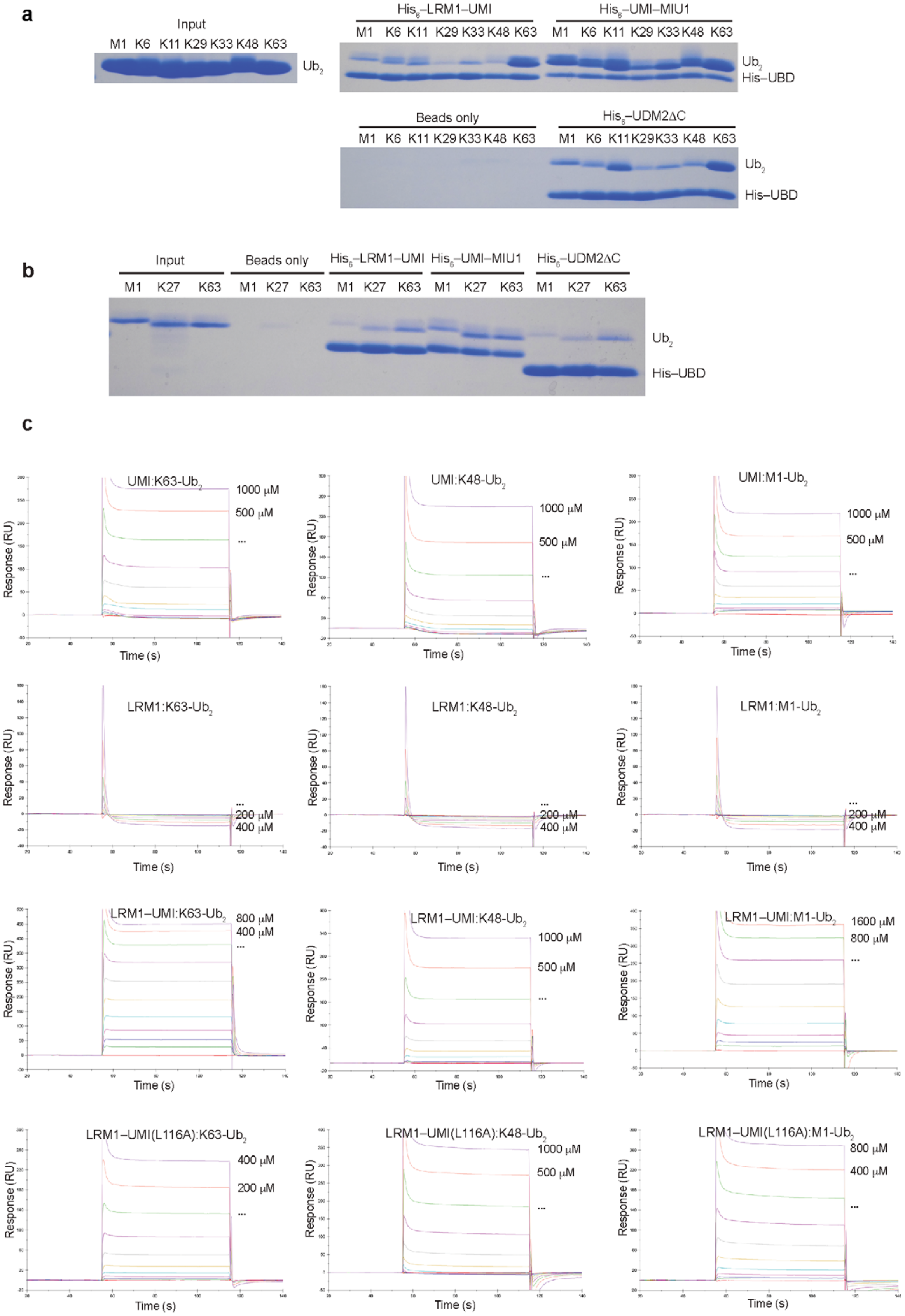

Supplementary Fig. 1 (continued)

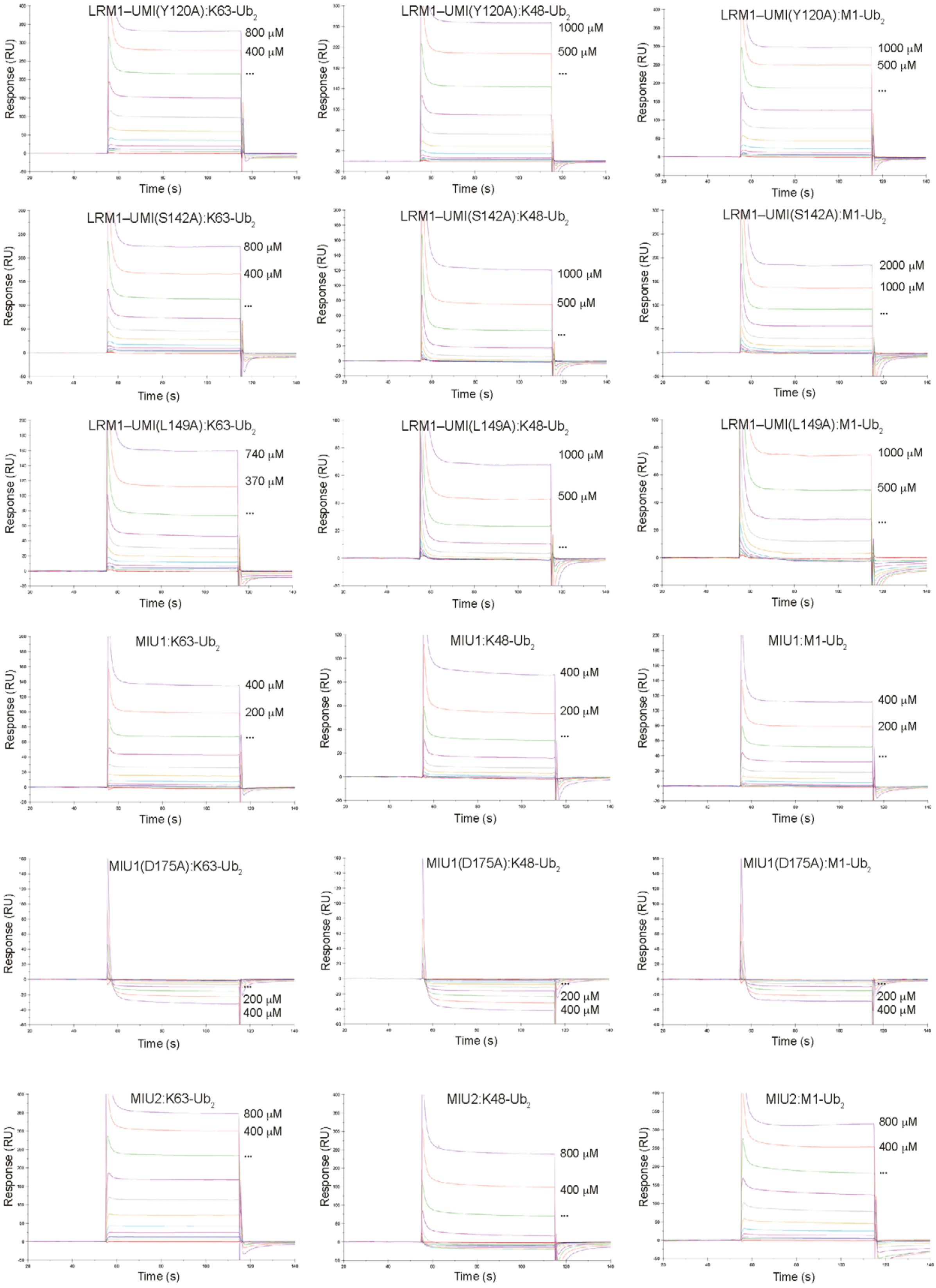

Supplementary Fig. 1 (continued)

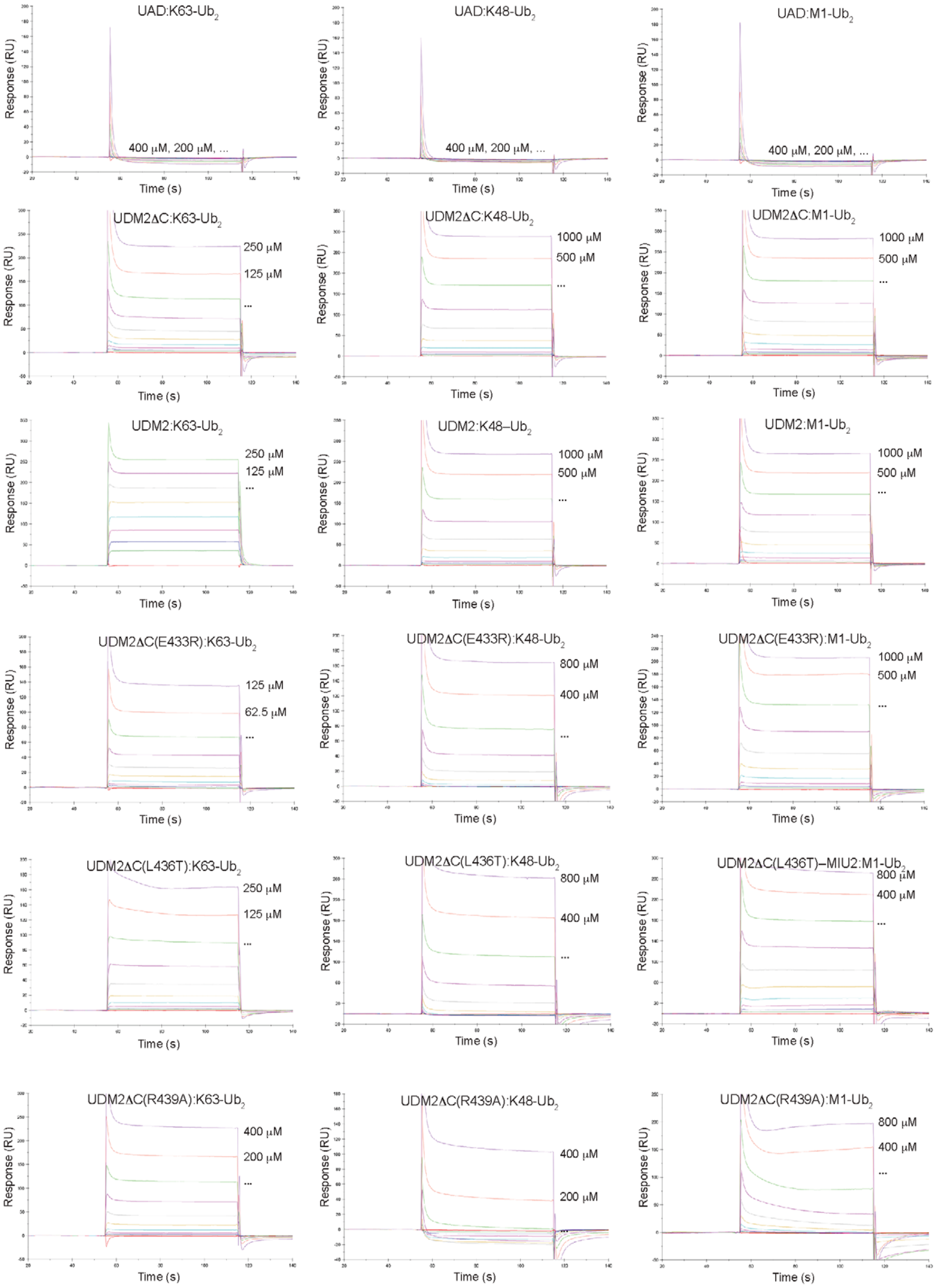

Supplementary Fig. 1 (continued)

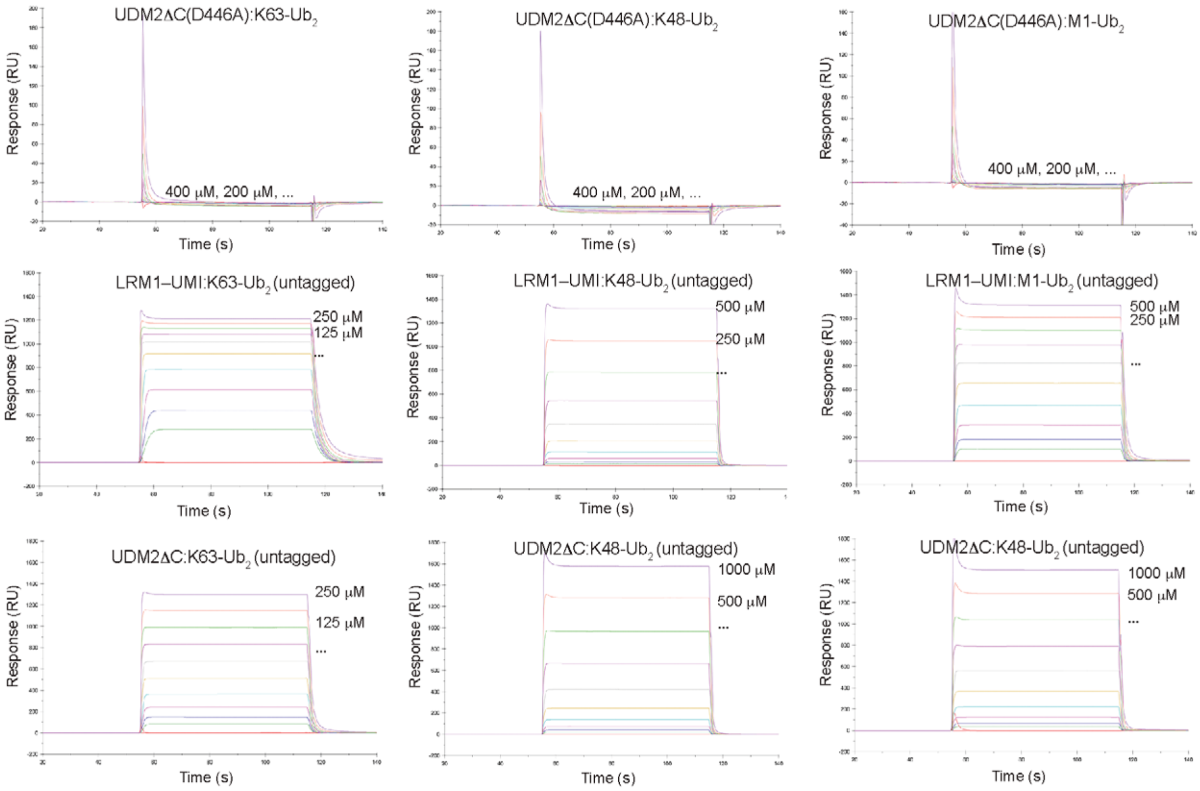

Supplementary Figure 1 Additional information of the molecular interaction analyses.
(a) Pull-down assays between UMI–MIU1, LRM1–UMI or UDM2ΔC of RNF168 and M1-, K6-, K11-, K29-, K33-, K48-, or K63-Ub_2. Bound proteins were analyzed by SDS-PAGE with Coomassie brilliant blue staining.
(b) Pull-down assays between UMI–MIU1, LRM1–UMI or UDM2ΔC of RNF168 and M1-, K27 or K63-Ub_2. Bound proteins were analyzed by SDS-PAGE with Coomassie brilliant blue staining.
(c) SPR sensorgrams that were used to calculate the affinities shown in Table 1. One representative sensorgram from three independent measurements is shown for each ligand–analyte pair.

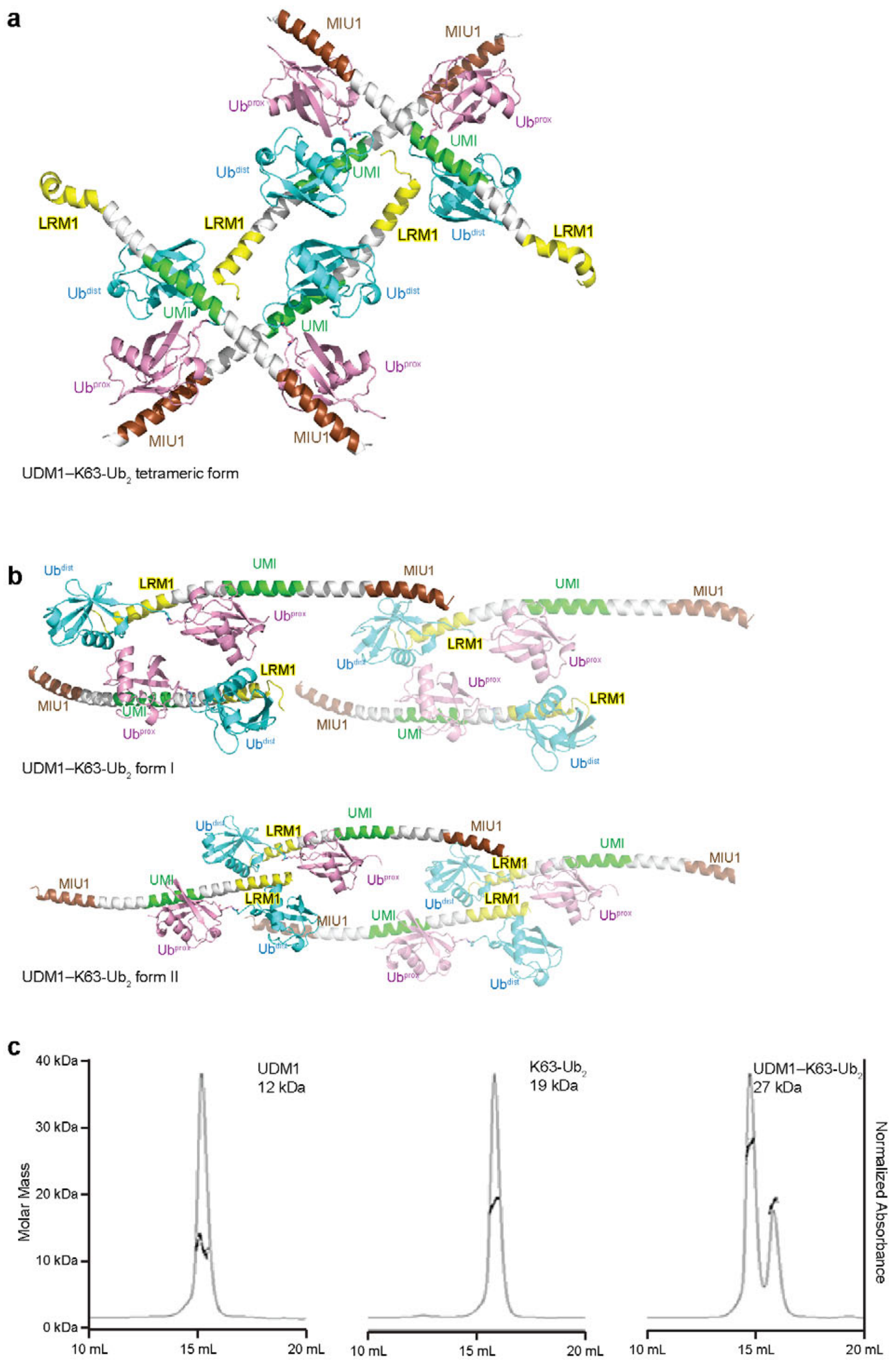

Supplementary Figure 2 Crystal packing of UDM1–K63-Ub$_2$.
The drawing scheme is the same as in Fig. 2.
(a) Two 2:2 UDM1–K63-Ub$_2$ complexes in the asymmetric unit in the tetrameric form crystal of UDM1–K63-Ub$_2$.
(b) Crystal packing of four 1:1 UDM1–K63-Ub$_2$ complexes in the form I and II crystals. The second asymmetric unit is displayed as translucent.
(c) SEC-MALS analyses of UDM1, K63-Ub$_2$ and a 2:1 mixture of UDM1 and K63-Ub$_2$. The molar mass calculated from the main peak in each sample is shown.

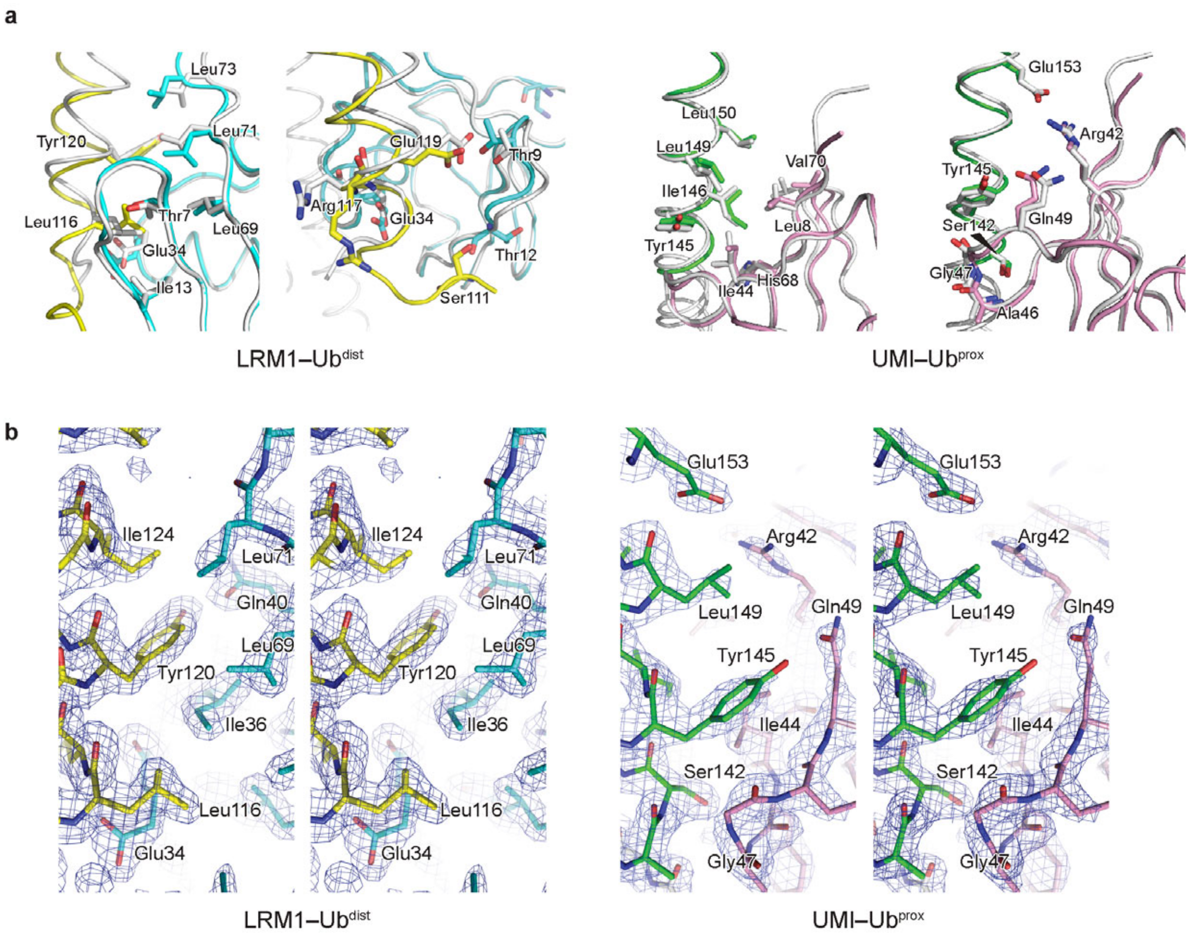

Supplementary Figure 3 Additional structural information of the UDM1–K63-Ub$_2$ complex.
The drawing scheme is the same as in Fig. 2.
(a) Comparison of the UMI–Ub^{prox} and LRM1–Ub^{dist} interfaces between the crystal forms I and II.
(b) Electron density around the interface between LRM1 and Ub^{dist} and that between UMI and Ub^{prox} ($2F_o$–F_c, contoured at 1.5σ level, stereo view).

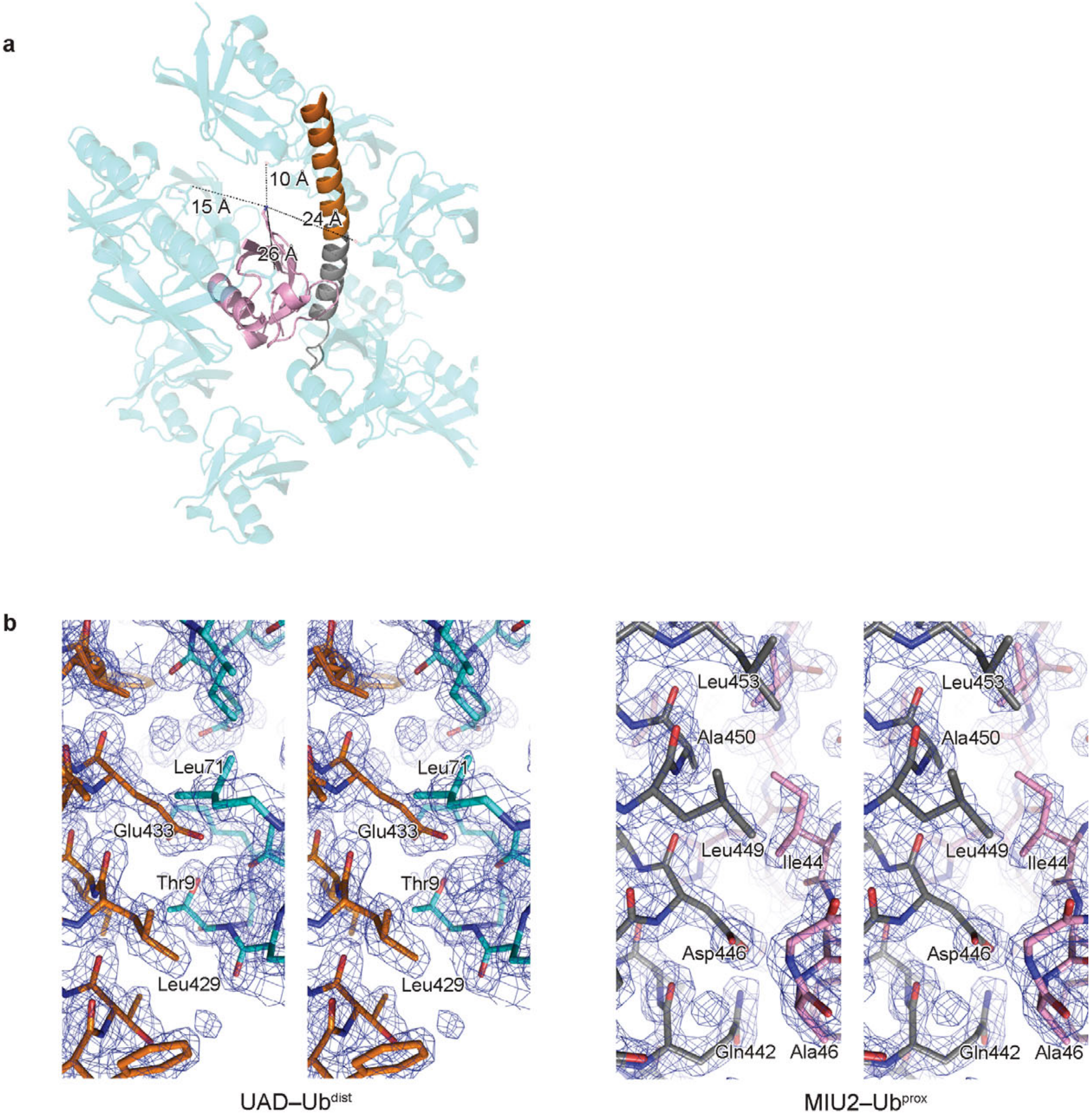

Supplementary Figure 4 Additional structural information of the UDM2ΔC–K63-Ub$_2$ complex. The drawing scheme is the same as in Fig. 3.

(a) Crystal packing of UDM2ΔC–Ub in the crystal of UDM2ΔC–K63-Ub$_2$. Neighboring Ub molecules are colored in translucent cyan. The distances between Lys63 of Ub^{prox} and Leu73 of three closest Ub molecules are shown.

(b) Electron density around the interface between UAD and Ub^{dist} and that between MIU2 and Ub^{prox} ($2F_o$–F_c, contoured at 1.5σ level, stereo view).

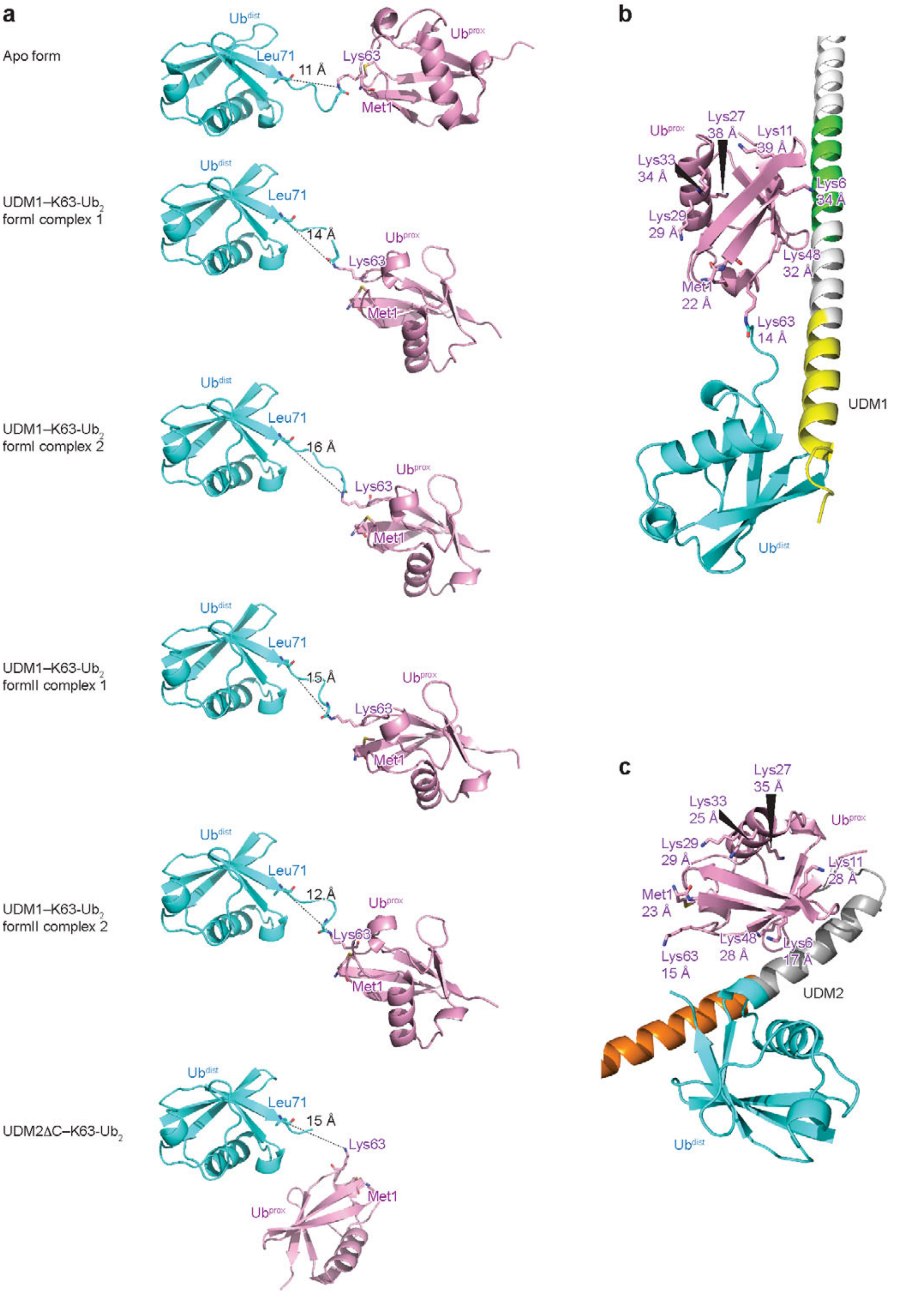

Supplementary Figure 5 Additional data on spacing between Ub^{dist} and Ub^{prox} of K63-Ub_2 bound to UDM1 or UDM2ΔC of RNF168.

The drawing scheme is the same as in Figs. 2 and 3.

(a) Distance between the Cα atom of Ub^{dist} Leu71 and the Nε atom of Ub^{prox} Lys63 in the apo (PDB 2JF5) or UDM1- or UDM2ΔC-bound K63-Ub_2.

(b, c) Positions of Met1 and seven lysine residues of Ub^{prox} in the UDM1–K63-Ub_2 (b) or UDM2ΔC–K63-Ub_2 (c) structure. The distances between the Cα atom of Ub^{dist} Leu71 and the Nε atom of Lys or main-chain N atom of Met1 in Ub^{prox} are shown.

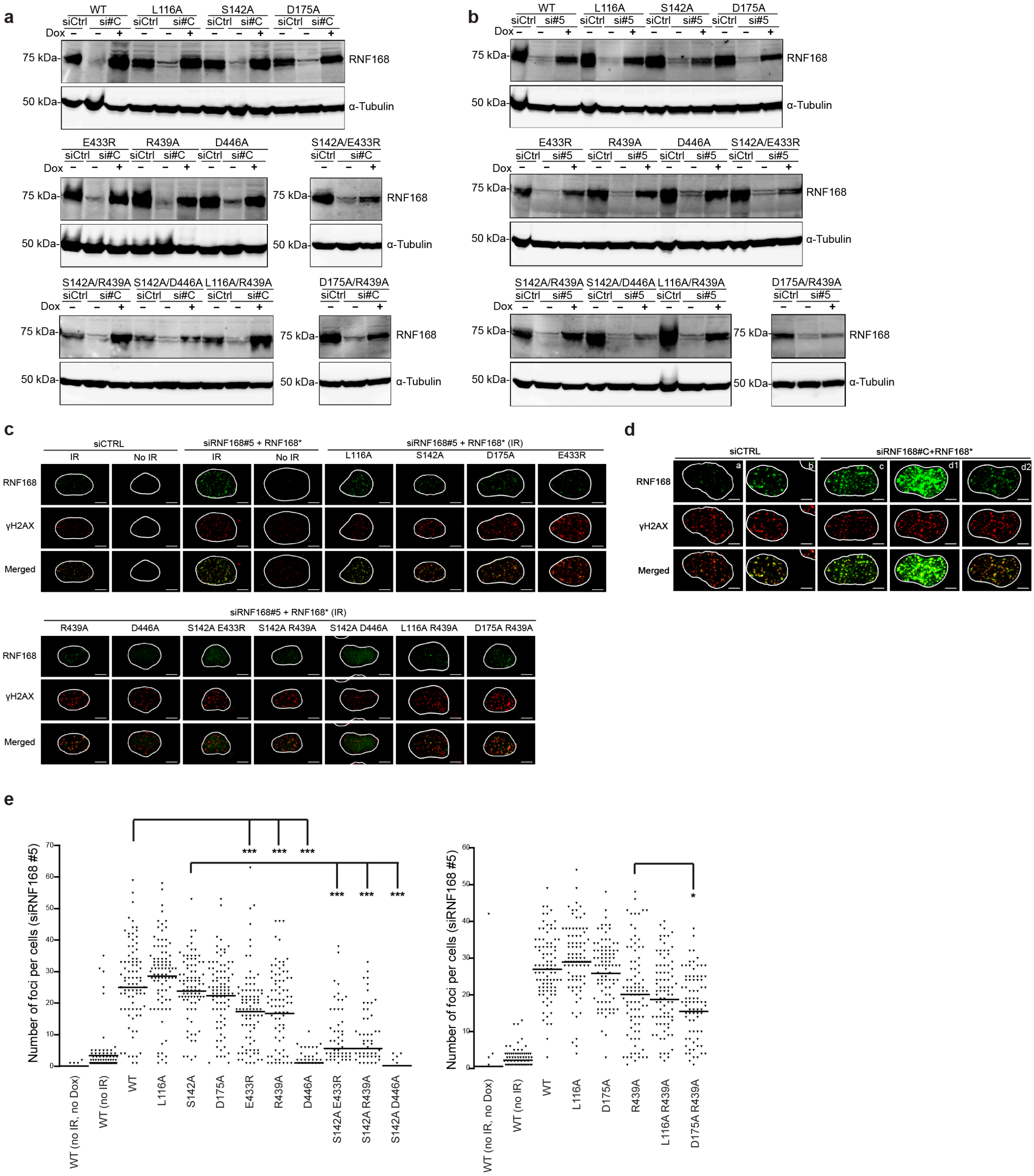

Supplementary Figure 6 Additional data on foci formation of wild-type or mutant RNF168 after gamma irradiation.

(a, b) siRNA-mediated knockdown of endogenous RNF168 and doxycycline-induced expression of RNF168 were confirmed by immunoblotting. Amounts of RNF168 and α-tubulin (loading control) were analyzed. siRNF168#C (a) or siRNF168#5 (b) was used for RNF168-knockdown.

(c) Representative images of immunofluorescence of siRNF168#5 transfected U2OS cells are shown. Wild-type or mutant RNF168 was expressed by doxycycline treatment. The nuclei visualized by DAPI staining were outlined with white borders. The scale bars indicate 5 μm. green; RNF168, red; γH2AX.

(d) Cells expressing high levels of doxycycline -induced RNF168. Endogenous RNF168 formed foci at DSB sites (panels a and b). siRNA-resistant RNF168 formed foci at DSB sites in siRNF168#C-transfected U2OS cells (panels c, d1 and d2). Confocal images of the panels d1 and d2 were acquired from the identical cell. Confocal images of the panels a, b, c and d1 were acquired under the same

detection sensitivity. Detection sensitivity used for acquiring RNF168 confocal image of the panel d2 was 10% of that of the panel d1 (γH2AX confocal images in the panels d1 and d2 were acquired using the same detection sensitivity). The cell in the panel (d) expressed a high level of RNF168. Cells with such high RNF168 expression were excluded from the analysis.
(e) Quantitation of (c). 90 cells were analyzed for each sample. The line represents the mean of the number of foci per cell. Significance is reported as the Kruskal–Wallis test (* $P < 0.05$; ** $P < 0.01$; *** $P < 0.001$).

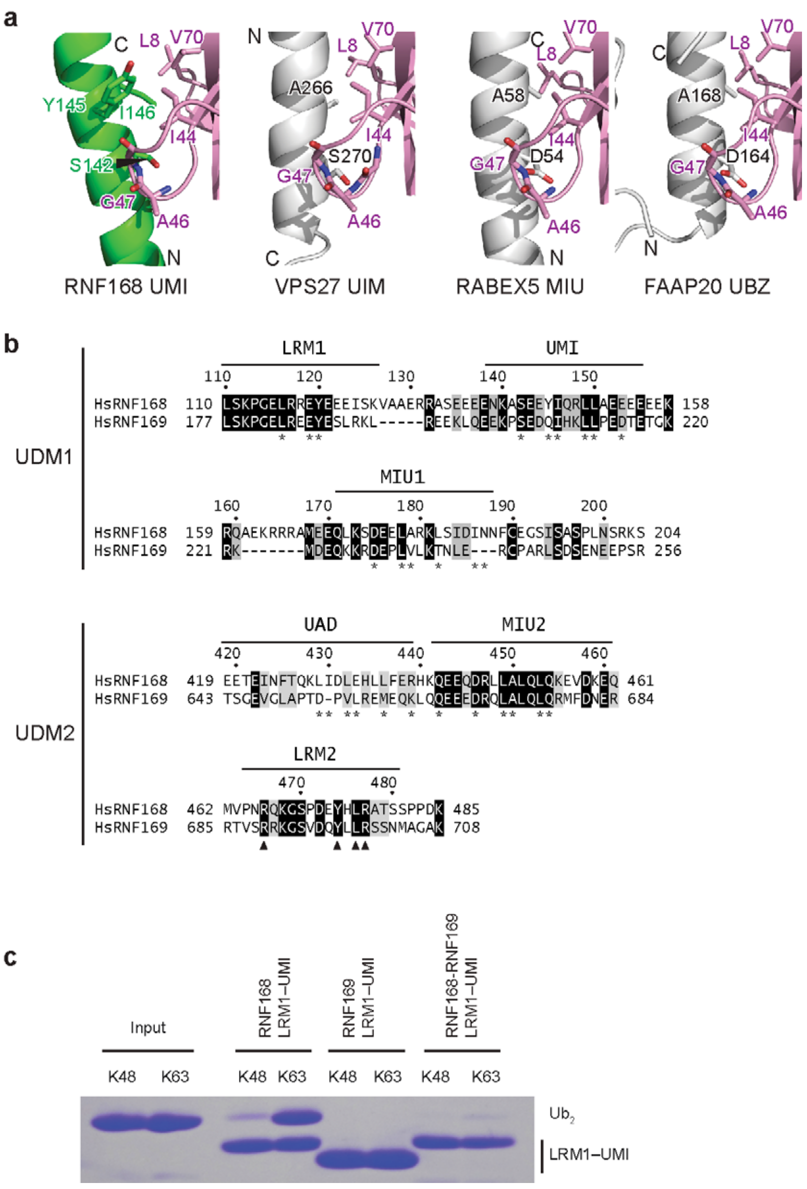

Supplementary Figure 7 Additional information about the UMI structure and RNF169 UDM1.
(a) Structural comparison of RNF168 UMI, VPS27 UIM, RABEX5 MIU and FAAP20 UBZ. The coloring scheme is the same as in Fig. 2. VPS27 UIM, RABEX5 MIU and FAAP20 UBZ are colored in white.
(b) Sequence alignment between human RNF168 and RNF169 sequences[56]. The drawing scheme is the same as in Fig. 2d, except that filled triangles indicate the LRM2 residues that were previously shown to affect the recruitment of RNF169[20].
(c) Pull-down assays between LRM1–UMI of RNF168, RNF169 or an RNF168-169 hybrid (residues 122–131 of RNF168 replaced by SLRKL) and K48- or K63-Ub_2. Bound proteins were analyzed by SDS-PAGE with Coomassie brilliant blue staining.

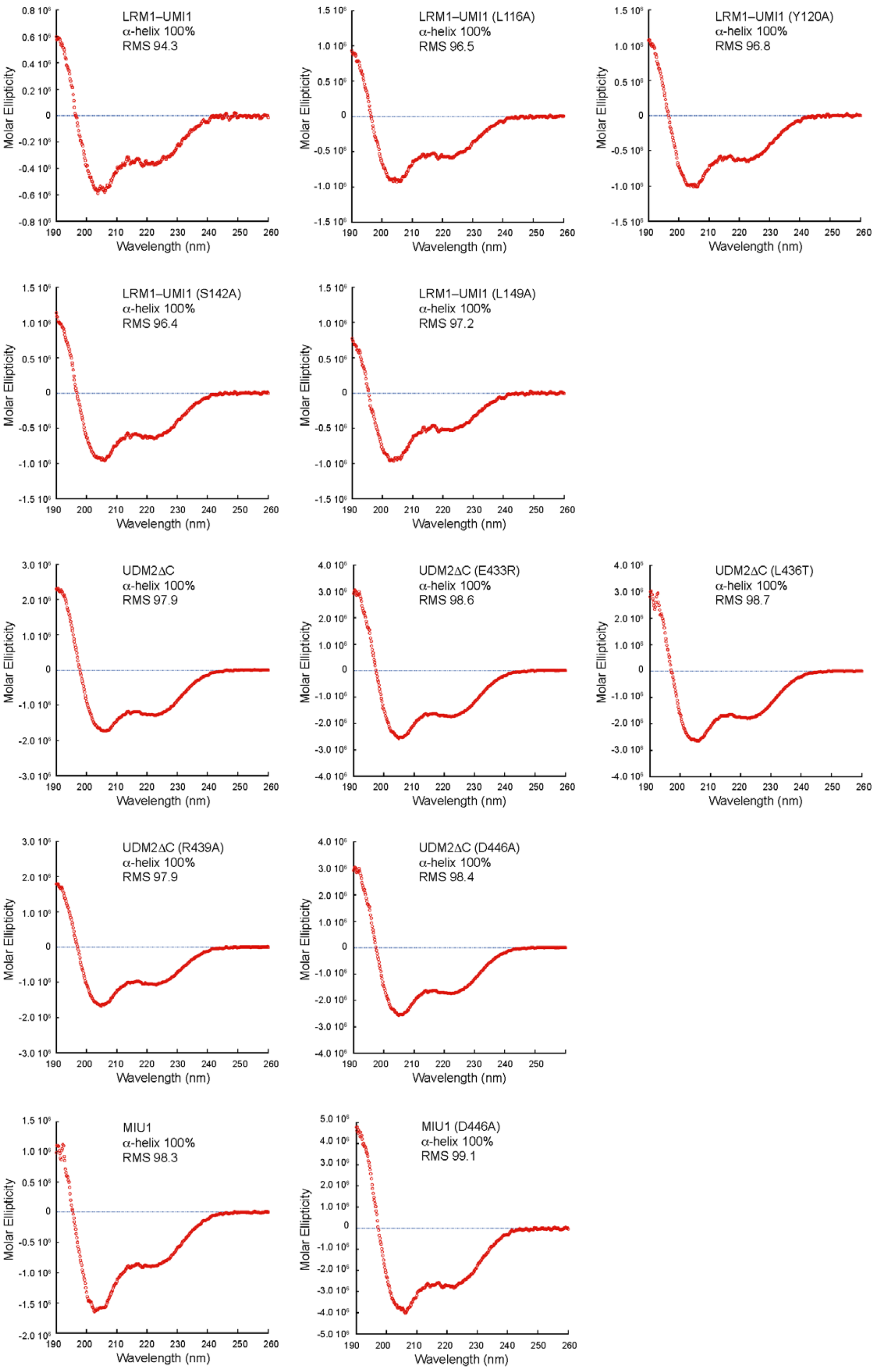

Supplementary Figure 8 CD spectra of wild-type and mutant LRM1–UMI, UDM2ΔC and MIU1.

Each plot represents the average of 4 measurements.

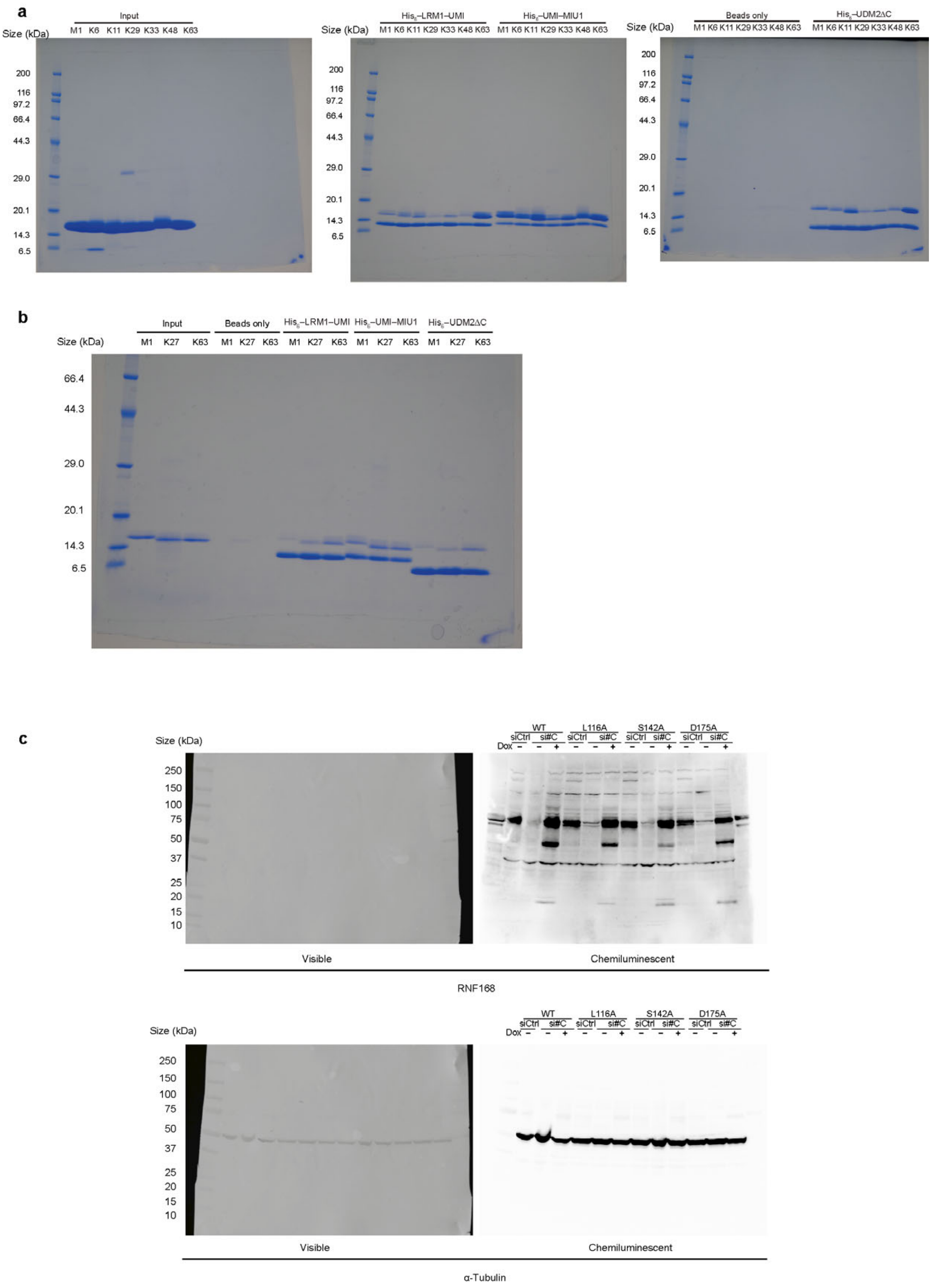

Supplementary Fig. 9 (continued)

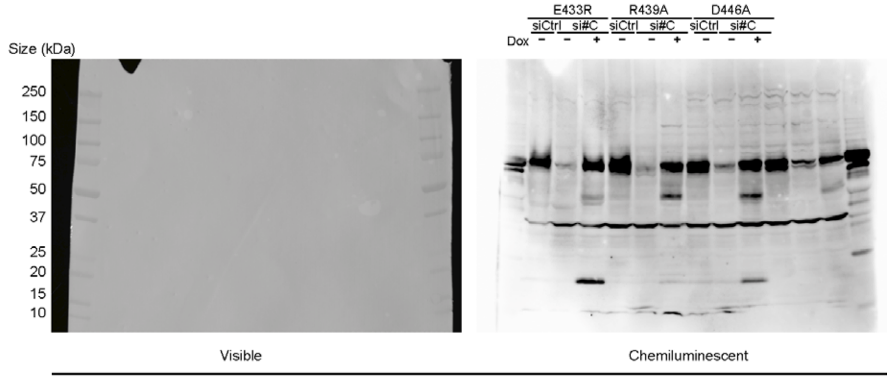

RNF168

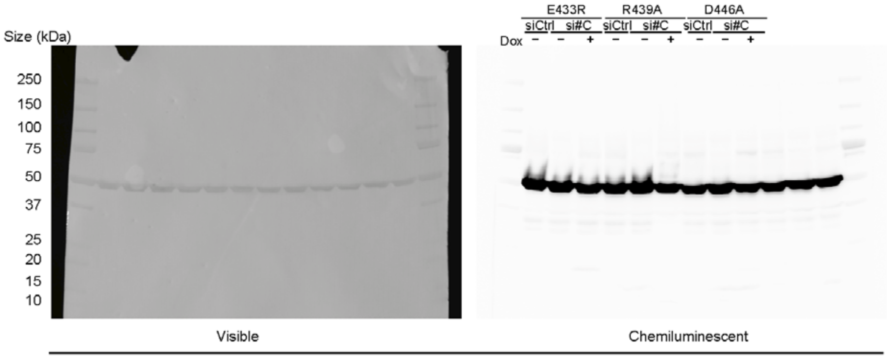

α-Tubulin

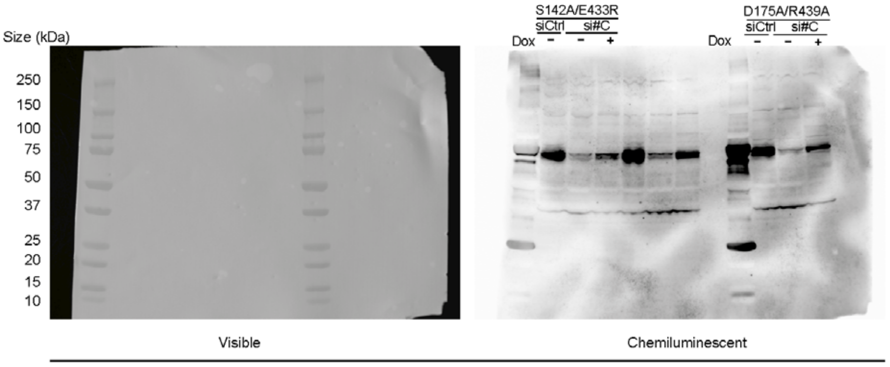

RNF168

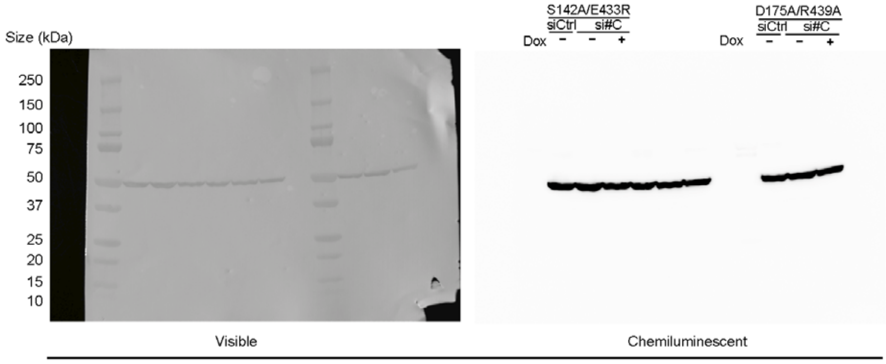

α-Tubulin

Supplementary Fig. 9 (continued)

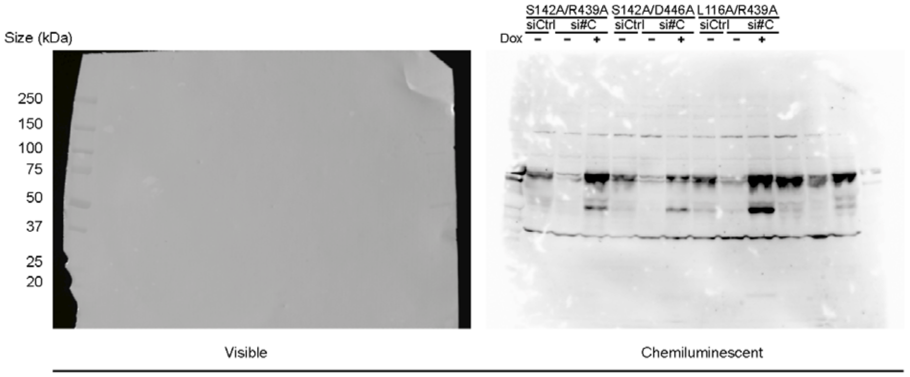

RNF168

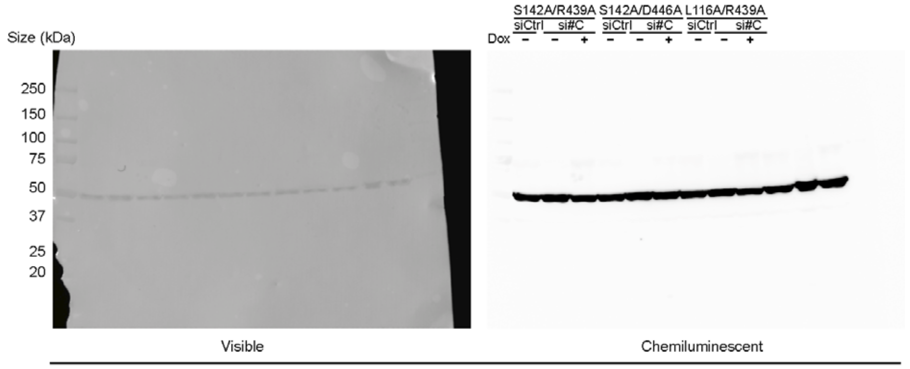

α-Tubulin

d

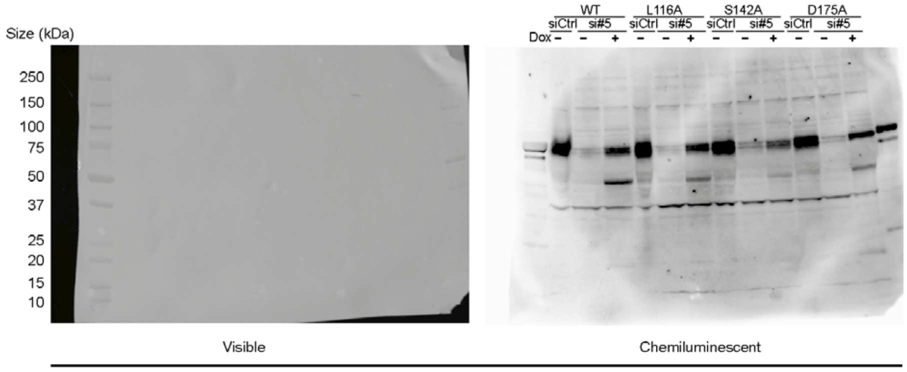

RNF168

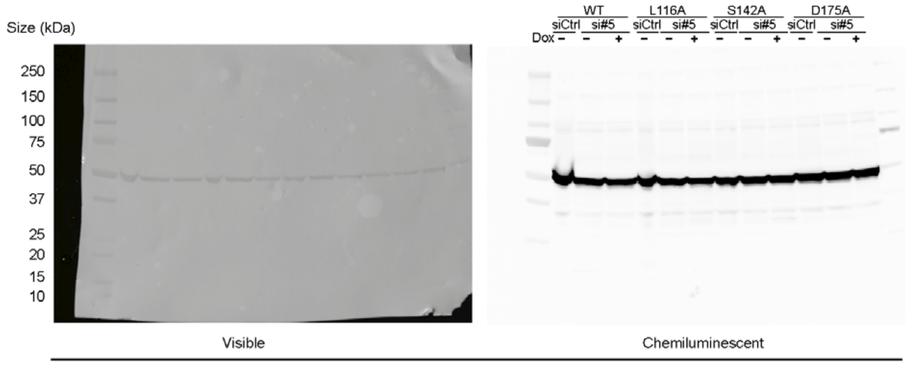

α-Tubulin

Supplementary Fig. 9 (continued)

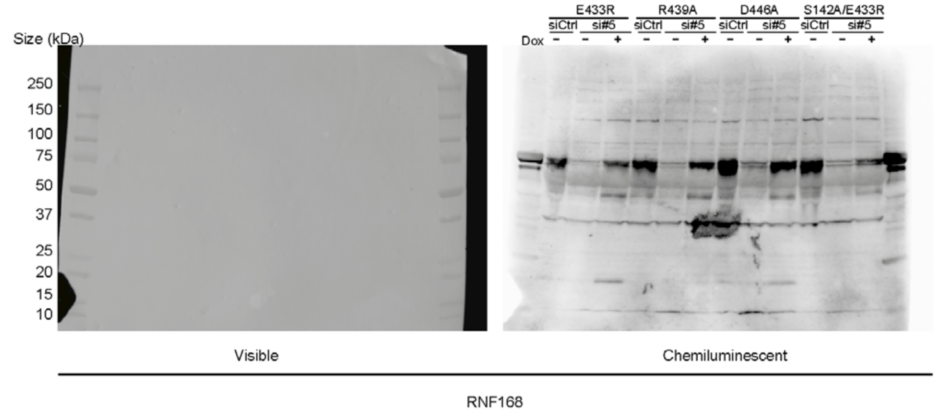

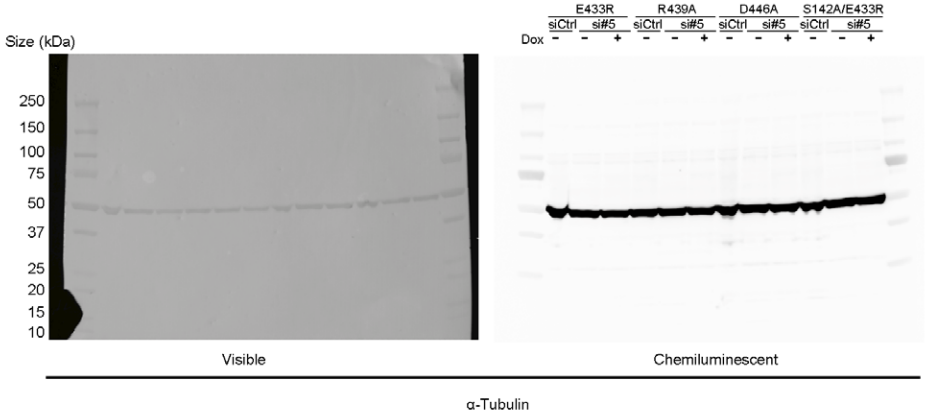

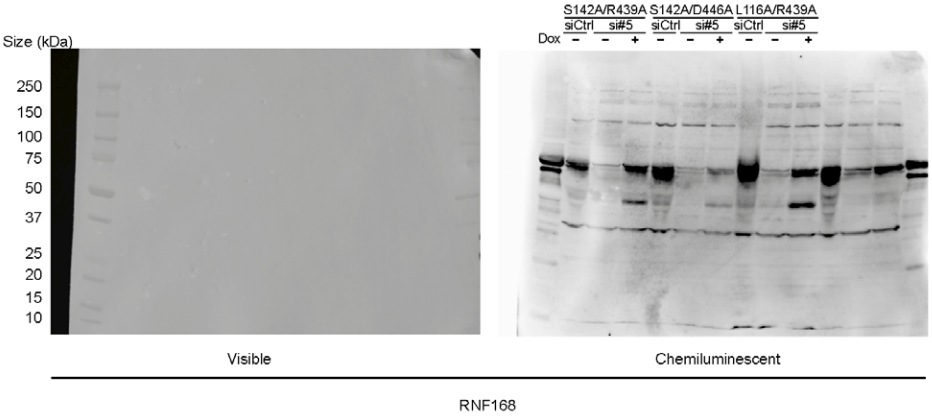

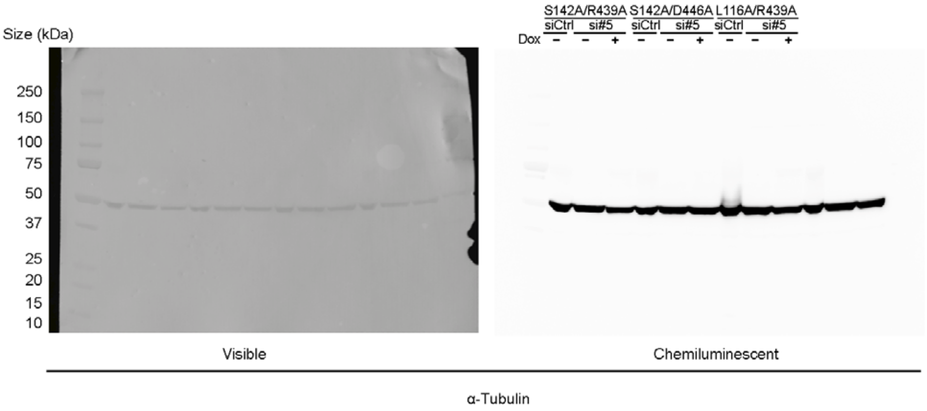

Supplementary Fig. 9 (continued)

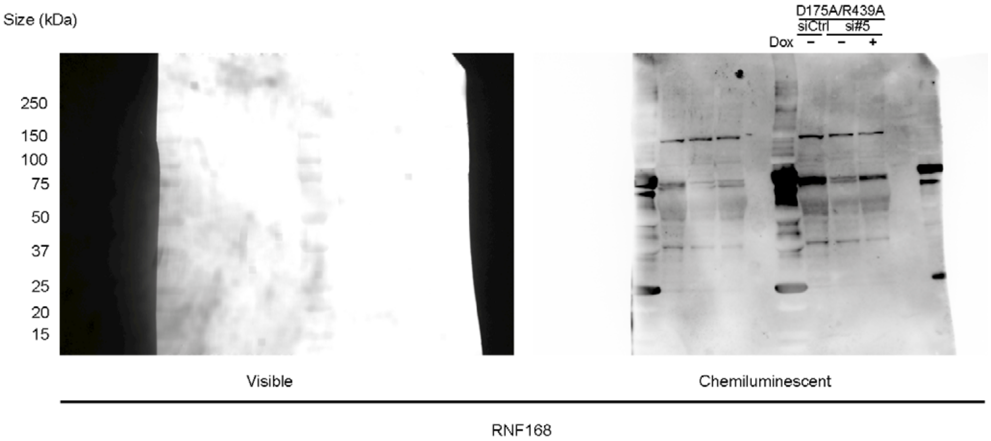

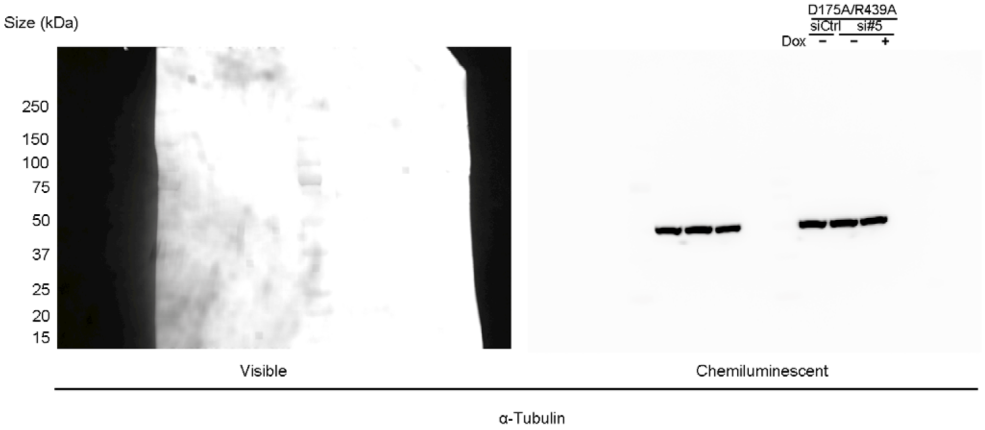

e

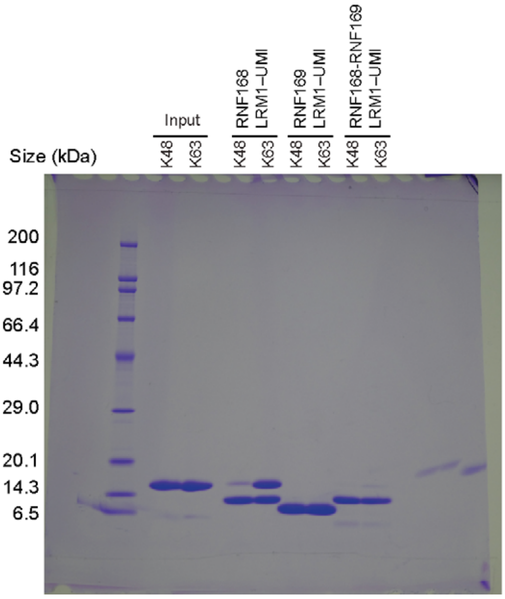

Supplementary Figure 9 Uncropped images of gels and blots.
(a) Original full-length gel images corresponding to Supplementary Fig. 1a
(b) Original full-length gel images corresponding to Supplementary Fig. 1b
(c) Original full-length blot images corresponding to Supplementary Fig. 6a
(d) Original full-length blot images corresponding to Supplementary Fig. 6b
(e) Original full-length gel images corresponding to Supplementary Fig. 7c
