# Supplementary material for: Structural insights into two distinct binding modules for Lys63-linked polyubiquitin chains in RNF168
Source: Nat Commun. 2018 Jan 12;9:170. doi: 10.1038/s41467-017-02345-y (PMC5766498; doi:10.1038/s41467-017-02345-y)
Supplement: Supplementary file 3 — Description of Additional Supplementary Files [file 41467_2017_2345_MOESM3_ESM.pdf]

Description of Supplementary Files

File Name: Supplementary Data 1
Description: Primer sequences used in this study.
